# The genome sequence of Clancy’s Rustic, *Caradrina kadenii* (Freyer, 1836)

**DOI:** 10.12688/wellcomeopenres.19286.1

**Published:** 2023-04-25

**Authors:** Gavin R. Broad, Natural History Museum Genome Acquisition Lab, Darwin Tree of Life Barcoding collective, Wellcome Sanger Institute Tree of Life programme, Wellcome Sanger Institute Scientific Operations: DNA Pipelines collective, Tree of Life Core Informatics collective, Darwin Tree of Life Consortium

**Affiliations:** 1 Natural History Museum, London, England, UK

**Keywords:** Caradrina kadenii, Clancy’s Rustic, genome sequence, chromosomal, Lepidoptera

## Abstract

We present a genome assembly from an individual male *Caradrina kadenii* (Clancy’s Rustic; Arthropoda; Insecta; Lepidoptera; Noctuidae). The genome sequence is 426.0 megabases in span. Most of the assembly is scaffolded into 31 chromosomal pseudomolecules, including the Z sex chromosome. The mitochondrial genome has also been assembled and is 15.4 kilobases in length.

## Species taxonomy

Eukaryota; Metazoa; Ecdysozoa; Arthropoda; Hexapoda; Insecta; Pterygota; Neoptera; Endopterygota; Lepidoptera; Glossata; Ditrysia; Noctuoidea; Noctuidae; Noctuinae; *Caradrina*; *Caradrina kadenii* (Freyer, 1836) (NCBI:txid987896).

## Background

One of nearly 190 species *of Caradrina*, *C. kadenii* is a species of the warmer areas of southern Europe and the Near East which has been expanding its range in Europe. First recorded in Britain in Kent in 2002, and then named Clancy’s rustic after the collector, *C. kadenii* rapidly colonised and is now widespread in southern England ( Waring *et al.*, 2017), particularly in Kent, where the sequenced individual was trapped. This spread is not restricted to Britain; the first Dutch record, for instance, was in 2006, followed by many more individuals ( de Vos *et al.*, 2010).

*Caradrina kadenii* is a fairly distinctive species amongst many superficially similar Xyleninae: it is basically greyish with a contrastingly dark reniform stigma (kidney mark). The larvae feed on a variety of herbaceous plants with the adults on the wing, and readily trapped at light, in two generations in Britain, from June to July, and then September to October. The second generation is larger.

This is the second published genome for a species of *Caradrina*, following the publication of a complete genome for *C. clavipalpis*, the Pale Mottled Willow ( Boyes & Boyes, 2022).

### Genome sequence report

**Figure 1.**
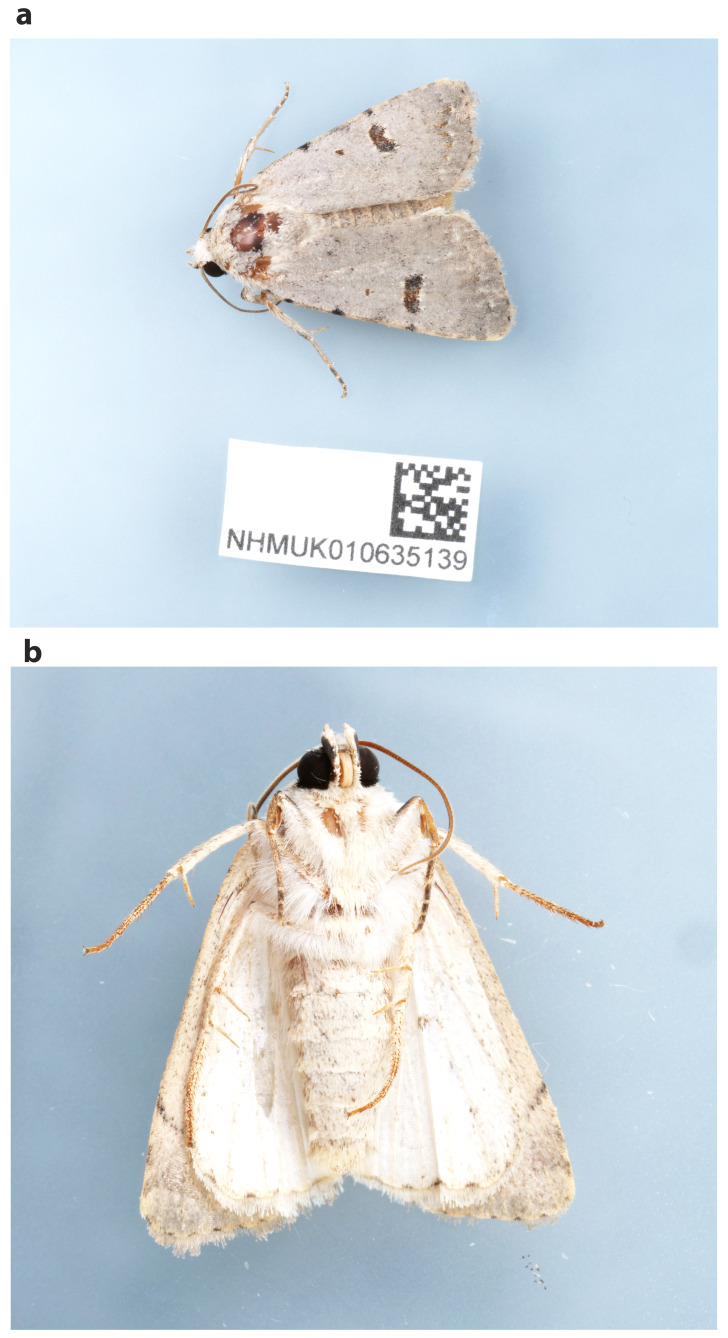
Photographs of the *Caradrina kadenii* (ilCarKade1) specimen used for genome sequencing: **a.** dorsal view, **b.** ventral view.

The genome was sequenced from one male *Caradrina kadenii* ( Figure 1) collected from Tonbridge, Kent, UK (latitude 50.25, longitude 1.64). A total of 36-fold coverage in Pacific Biosciences single-molecule HiFi long reads and 98-fold coverage in 10X Genomics read clouds were generated. Primary assembly contigs were scaffolded with chromosome conformation Hi-C data. Manual assembly curation corrected eight missing joins or mis-joins, reducing the scaffold number by 2.78%.

**Figure 2.**
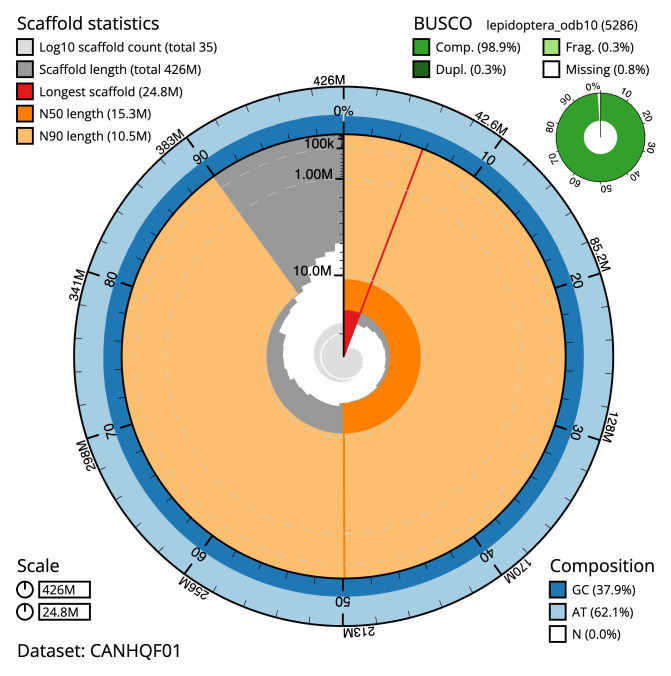
Genome assembly of *Caradrina kadenii*, ilCarKade1.1: metrics. The BlobToolKit Snailplot shows N50 metrics and BUSCO gene completeness. The main plot is divided into 1,000 size-ordered bins around the circumference with each bin representing 0.1% of the 426,002,513 bp assembly. The distribution of scaffold lengths is shown in dark grey with the plot radius scaled to the longest scaffold present in the assembly (24,750,785 bp, shown in red). Orange and pale-orange arcs show the N50 and N90 scaffold lengths (15,330,137 and 10,541,016 bp), respectively. The pale grey spiral shows the cumulative scaffold count on a log scale with white scale lines showing successive orders of magnitude. The blue and pale-blue area around the outside of the plot shows the distribution of GC, AT and N percentages in the same bins as the inner plot. A summary of complete, fragmented, duplicated and missing BUSCO genes in the lepidoptera_odb10 set is shown in the top right. An interactive version of this figure is available at https://blobtoolkit.genomehubs.org/view/ilCarKade1.1/dataset/CANHQF01/snail.

The final assembly has a total length of 426.0 Mb in 35 sequence scaffolds with a scaffold N50 of 15.3 Mb ( Table 1). Most (99.98%) of the assembly sequence was assigned to 31 chromosomal-level scaffolds, representing 30 autosomes, and the Z sex chromosome. Chromosome-scale scaffolds confirmed by the Hi-C data are named in order of size ( Figure 2– Figure 5; Table 2). While not fully phased, the assembly deposited is of one haplotype. Contigs corresponding to the second haplotype have also been deposited. The mitochondrial genome was also assembled and can be found as a contig within the multifasta file of the genome submission.

**Table 1.** Genome data for *Caradrina kadenii*, ilCarKade1.1.

Project accession data		
Assembly identifier	ilCarKade1.1	
Species	*Caradrina kadenii*	
Specimen	ilCarKade1	
NCBI taxonomy ID	987896	
BioProject	PRJEB55745	
BioSample ID	SAMEA8534280	
Isolate information	ilCarKade1, male: abdomen (genome sequencing), head and thorax (Hi-C scaffolding)	
Assembly metrics *		*Benchmark*
Consensus quality (QV)	69.4	*≥ 50*
*k*-mer completeness	100%	*≥ 95%*
BUSCO **	C:98.9%[S:98.6%,D:0.3%], F:0.3%,M:0.8%,n:5,286	*C ≥ 95%*
Percentage of assembly mapped to chromosomes	99.98%	*≥ 95%*
Sex chromosomes	Z chromosome	*localised* *homologous pairs*
Organelles	Mitochondrial genome assembled	*complete single* *alleles*
Raw data accessions		
PacificBiosciences SEQUEL II	ERR10168732	
10X Genomics Illumina	ERR10149561–ERR10149564	
Hi-C Illumina	ERR10149565	
Genome assembly		
Assembly accession	GCA_947462355.1	
*Accession of alternate haplotype*	GCA_947462605.1	
Span (Mb)	426.0	
Number of contigs	40	
Contig N50 length (Mb)	15.3	
Number of scaffolds	35	
Scaffold N50 length (Mb)	15.3	
Longest scaffold (Mb)	24.8	

* Assembly metric benchmarks are adapted from column VGP-2020 of “Table 1: Proposed standards and metrics for defining genome assembly quality” from ( Rhie *et al.*, 2021). ** BUSCO scores based on the lepidoptera_odb10 BUSCO set using v5.3.2. C = complete [S = single copy, D = duplicated], F = fragmented, M = missing, n = number of orthologues in comparison. A full set of BUSCO scores is available at https://blobtoolkit.genomehubs.org/view/ilCarKade1.1/dataset/CANHQF01/busco.

**Figure 3.**
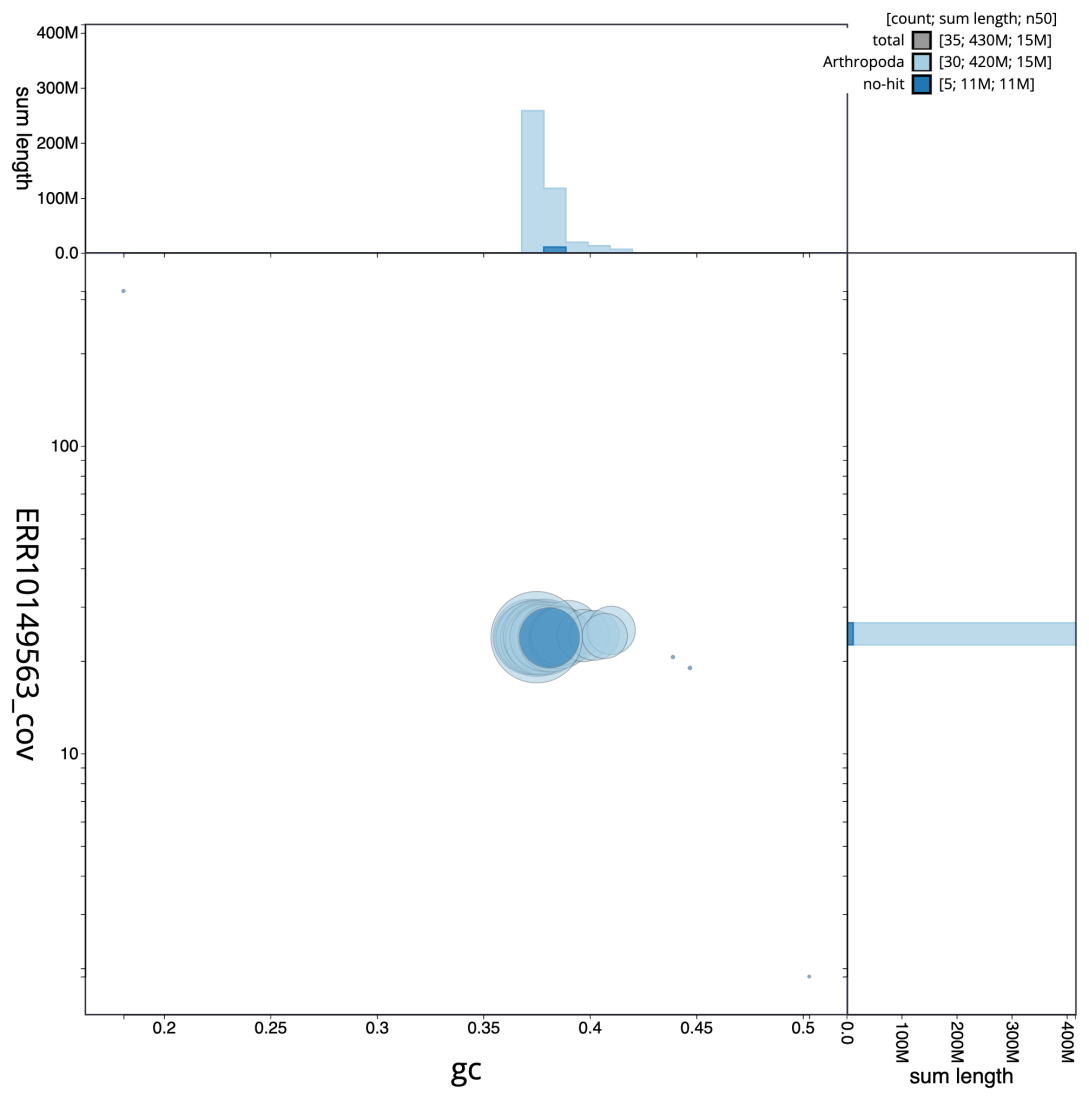
Genome assembly of *Caradrina kadenii*, ilCarKade1.1: BlobToolKit GC-coverage plot. Scaffolds are coloured by phylum. Circles are sized in proportion to scaffold length. Histograms show the distribution of scaffold length sum along each axis. An interactive version of this figure is available at https://blobtoolkit.genomehubs.org/view/ilCarKade1.1/dataset/CANHQF01/blob.

**Figure 4.**
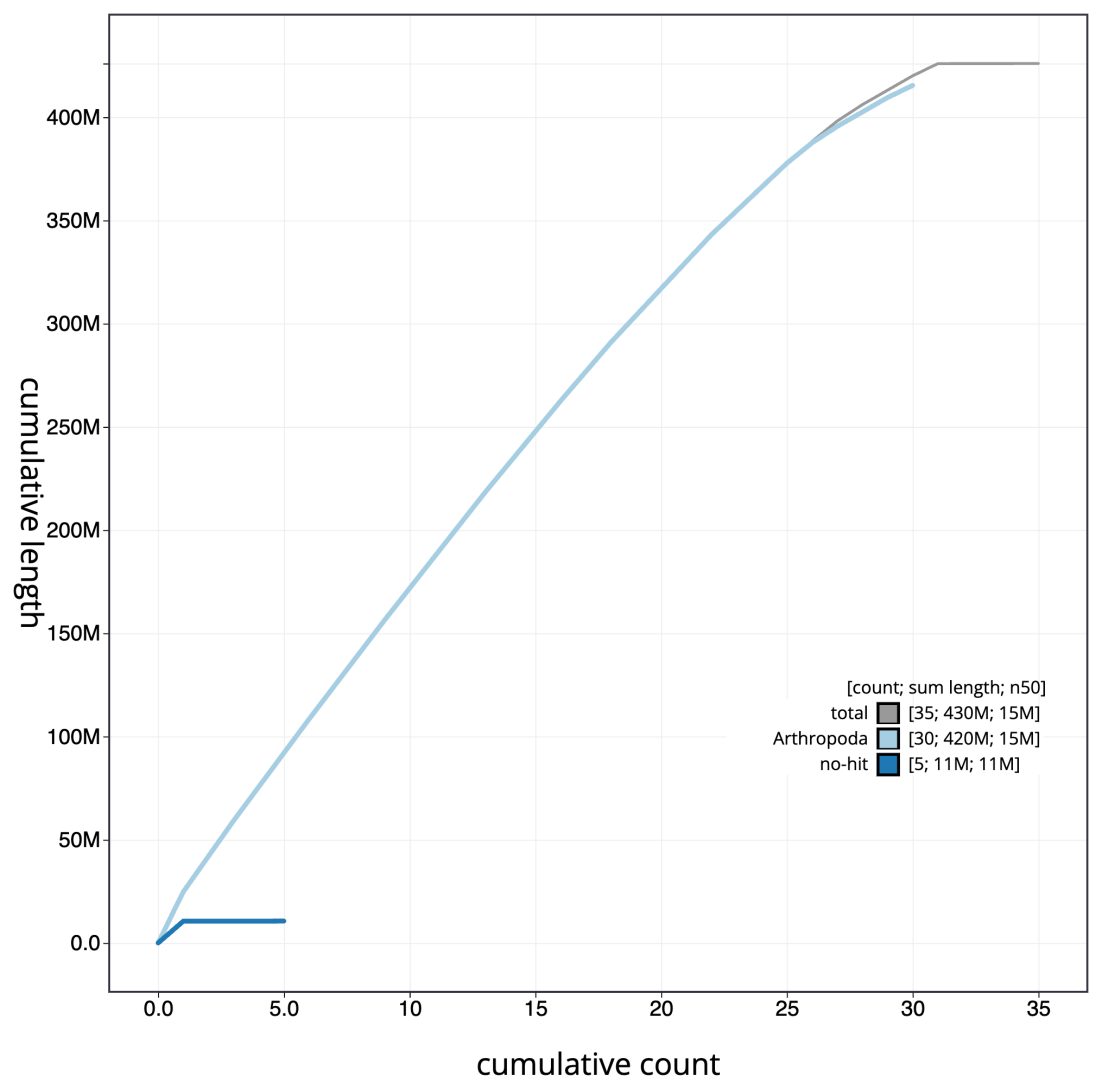
Genome assembly of *Caradrina kadenii*, ilCarKade1.1: BlobToolKit cumulative sequence plot. The grey line shows cumulative length for all scaffolds. Coloured lines show cumulative lengths of scaffolds assigned to each phylum using the buscogenes taxrule. An interactive version of this figure is available at https://blobtoolkit.genomehubs.org/view/ilCarKade1.1/dataset/CANHQF01/cumulative.

**Figure 5.**
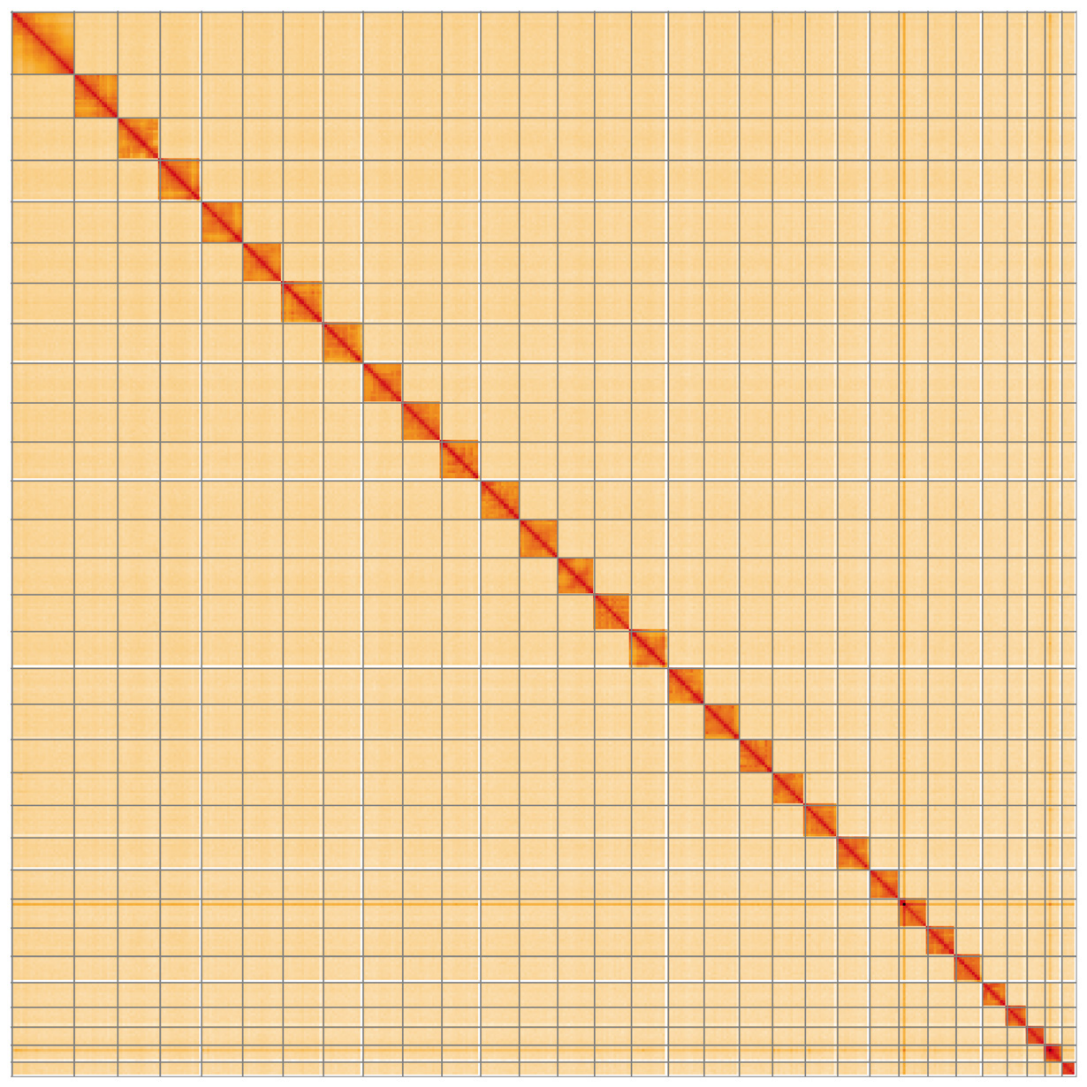
Genome assembly of *Caradrina kadenii*, ilCarKade1.1: Hi-C contact map of the ilCarKade1.1 assembly, visualised using HiGlass. Chromosomes are shown in order of size from left to right and top to bottom. An interactive version of this figure may be viewed at https://genome-note-higlass.tol.sanger.ac.uk/l/?d=DPf8NmFFR9qji4FeSDw70g.

**Table 2.** Chromosomal pseudomolecules in the genome assembly of *Caradrina kadenii*, ilCarKade1.

INSDC accession	Chromosome	Size (Mb)	GC%
OX381673.1	1	17.5	37.5
OX381674.1	2	16.96	37.9
OX381675.1	3	16.59	37.8
OX381676.1	4	16.38	37.2
OX381677.1	5	16.22	37.4
OX381678.1	6	16.12	37.6
OX381679.1	7	15.91	37.3
OX381680.1	8	15.88	37.4
OX381681.1	9	15.57	37.7
OX381682.1	10	15.53	37.2
OX381683.1	11	15.52	37.3
OX381684.1	12	15.33	37.5
OX381685.1	13	14.77	37.6
OX381686.1	14	14.76	37.6
OX381687.1	15	14.73	37.7
OX381688.1	16	14.28	37.9
OX381689.1	17	14.09	38
OX381690.1	18	13.34	38.3
OX381691.1	19	13.06	38.2
OX381692.1	20	12.97	37.8
OX381693.1	21	12.84	38.2
OX381694.1	22	11.64	38.1
OX381695.1	23	11.63	39
OX381696.1	24	11.35	38.6
OX381697.1	25	10.54	38.1
OX381698.1	26	9.87	38.6
OX381699.1	27	7.94	39.7
OX381700.1	28	7.22	40.2
OX381701.1	29	6.76	41
OX381702.1	30	5.89	40.7
OX381672.1	Z	24.75	37.5
OX381703.1	MT	0.02	18.3

The estimated Quality Value (QV) of the final assembly is 69.4 with a *k*-mer completeness of 100%, and the assembly has a BUSCO v5.3.2 ( Manni *et al.*, 2021) completeness of 98.9%% (single = 98.6%, duplicated = 0.3%), using the lepidoptera_odb10 reference set ( *n* = 5,286).

## Methods

### Sample acquisition and nucleic acid extraction

A male *Caradrina kadenii* (ilCarKade1) was collected from a garden in Tonbridge, Kent, UK (latitude 50.25, longitude 1.64) on 20 August 2020, using a light trap. The specimen was collected and identified by Gavin Broad (Natural History Museum), and preserved in liquid nitrogen.

DNA was extracted at the Tree of Life laboratory, Wellcome Sanger Institute (WSI). The ilCarKade1 sample was weighed and dissected on dry ice with head and thorax tissue set aside for Hi-C sequencing. Abdomen tissue was disrupted using a Nippi Powermasher fitted with a BioMasher pestle. High molecular weight (HMW) DNA was extracted using the Qiagen MagAttract HMW DNA extraction kit. Low molecular weight DNA was removed from a 20-ng aliquot of extracted DNA using the 0.8X AMpure XP purification kit prior to 10X Chromium sequencing; a minimum of 50 ng DNA was submitted for 10X sequencing. HMW DNA was sheared into an average fragment size of 12–20 kb in a Megaruptor 3 system with speed setting 30. Sheared DNA was purified by solid-phase reversible immobilisation using AMPure PB beads with a 1.8X ratio of beads to sample to remove the shorter fragments and concentrate the DNA sample. The concentration of the sheared and purified DNA was assessed using a Nanodrop spectrophotometer and Qubit Fluorometer and Qubit dsDNA High Sensitivity Assay kit. Fragment size distribution was evaluated by running the sample on the FemtoPulse system.

### Sequencing

Pacific Biosciences HiFi circular consensus and 10X Genomics read cloud DNA sequencing libraries were constructed according to the manufacturers’ instructions. DNA sequencing was performed by the Scientific Operations core at the WSI on Pacific Biosciences SEQUEL II (HiFi) and Illumina NovaSeq 6000 (10X) instruments. Hi-C data were also generated from head and thorax tissue of ilCarKade1 using the Arima v2 kit and sequenced on the Illumina NovaSeq 6000 instrument.

### Genome assembly, curation and evaluation

Assembly was carried out with Hifiasm ( Cheng *et al.*, 2021) and haplotypic duplication was identified and removed with purge_dups ( Guan *et al.*, 2020). One round of polishing was performed by aligning 10X Genomics read data to the assembly with Long Ranger ALIGN, calling variants with FreeBayes ( Garrison & Marth, 2012). The assembly was then scaffolded with Hi-C data ( Rao *et al.*, 2014) using YaHS ( Zhou *et al.*, 2023). The assembly was checked for contamination as described previously ( Howe *et al.*, 2021). Manual curation was performed using HiGlass ( Kerpedjiev *et al.*, 2018) and Pretext ( Harry, 2022). The mitochondrial genome was assembled using MitoHiFi ( Uliano-Silva *et al.*, 2022), which performed annotation using MitoFinder ( Allio *et al.*, 2020). To evaluate the assembly, MerquryFK was used to estimate consensus quality (QV) scores and *k*-mer completeness ( Rhie *et al.*, 2020). The genome was analysed and BUSCO scores ( Manni *et al.*, 2021; Simão *et al.*, 2015) were calculated within the BlobToolKit environment ( Challis *et al.*, 2020). Table 3 contains a list of software tool versions and sources.

**Table 3.** Software tools: versions and sources.

Software tool	Version	Source
BlobToolKit	4.0.7	https://github.com/blobtoolkit/blobtoolkit
BUSCO	5.3.2	https://gitlab.com/ezlab/busco
Hifiasm	0.16.1-r375	https://github.com/chhylp123/hifiasm
HiGlass	1.11.6	https://github.com/higlass/higlass
Merqury	MerquryFK	https://github.com/thegenemyers/MERQURY.FK
MitoHiFi	2	https://github.com/marcelauliano/MitoHiFi
PretextView	0.2	https://github.com/wtsi-hpag/PretextView
purge_dups	1.2.3	https://github.com/dfguan/purge_dups
YaHS	yahs-1.1.91eebc2	https://github.com/c-zhou/yahs

### Ethics and compliance issues

The materials that have contributed to this genome note have been supplied by a Darwin Tree of Life Partner. The submission of materials by a Darwin Tree of Life Partner is subject to the Darwin Tree of Life Project Sampling Code of Practice. By agreeing with and signing up to the Sampling Code of Practice, the Darwin Tree of Life Partner agrees they will meet the legal and ethical requirements and standards set out within this document in respect of all samples acquired for, and supplied to, the Darwin Tree of Life Project. All efforts are undertaken to minimise the suffering of animals used for sequencing. Each transfer of samples is further undertaken according to a Research Collaboration Agreement or Material Transfer Agreement entered into by the Darwin Tree of Life Partner, Genome Research Limited (operating as the Wellcome Sanger Institute), and in some circumstances other Darwin Tree of Life collaborators.

## Data Availability

European Nucleotide Archive: *Caradrina kadenii.* Accession number PRJEB55745; https://identifiers.org/ena.embl/PRJEB55745 ( Wellcome Sanger Institute, 2022) The genome sequence is released openly for reuse. The *Caradrina kadenii* genome sequencing initiative is part of the Darwin Tree of Life (DToL) project. All raw sequence data and the assembly have been deposited in INSDC databases. The genome will be annotated using available RNA-Seq data and presented through the Ensembl pipeline at the European Bioinformatics Institute. Raw data and assembly accession identifiers are reported in Table 1. Members of the Natural History Museum Genome Acquisition Lab are listed here: https://doi.org/10.5281/zenodo.4790042. Members of the Darwin Tree of Life Barcoding collective are listed here: https://doi.org/10.5281/zenodo.4893703. Members of the Wellcome Sanger Institute Tree of Life programme are listed here: https://doi.org/10.5281/zenodo.4783585. Members of Wellcome Sanger Institute Scientific Operations: DNA Pipelines collective are listed here: https://doi.org/10.5281/zenodo.4790455. Members of the Tree of Life Core Informatics collective are listed here: https://doi.org/10.5281/zenodo.5013541. Members of the Darwin Tree of Life Consortium are listed here: https://doi.org/10.5281/zenodo.4783558.
