# Supplementary material for: The genome sequence of Clancy’s Rustic, Caradrina kadenii (Freyer, 1836)
Source: Wellcome Open Res. 2023 Apr 25;8:187. [Version 1] doi: 10.12688/wellcomeopenres.19286.1 (PMC10565420; doi:10.12688/wellcomeopenres.19286.1)
Supplement: Peer review history

# Reviewer report: Reviewer response for version 1

**DOI:** 10.21956/wellcomeopenres.21369.r67484

**Published:** 2023-10-12

**Authors:** Marko Mutanen

**Affiliations:** 1 University of Oulu, Oulu, Finland

This works provides a high-quality reference genome for a Noctuid moth Caradrina kadenii, a highly expansive moth species in Europe. A reference genome for the closely related C. clavipalpis (a semi-synanthropic species) has been generated earlier. No comparison to this species is made in this connection. The genome with chromosomal-level assemblies is of high quality according to the provided measures. Illustrations of the sequenced female specimen are given. Features of the genome are illustrated in several useful ways. The work is technically sound.

 It is stated that the species is morphologically a fairly distinctive species. This may be true in the area of collection, but I think several quite similar-looking species occur elsewhere, also in Europe, including C. montana and C. albina. This is a very minor detail, though.

 This work is an important contribution which helps us to understand e.g. Lepidoptera phylogenomics, but is potentially useful in many other areas as well.

Are sufficient details of methods and materials provided to allow replication by others?

Yes

Is the rationale for creating the dataset(s) clearly described?

Yes

Are the datasets clearly presented in a useable and accessible format?

Yes

Are the protocols appropriate and is the work technically sound?

Yes

Reviewer Expertise:

Insect phylogenomics, molecular taxonomy, biodiversity

I confirm that I have read this submission and believe that I have an appropriate level of expertise to confirm that it is of an acceptable scientific standard.

# Reviewer report: Reviewer response for version 1

**DOI:** 10.21956/wellcomeopenres.21369.r67486

**Published:** 2023-10-10

**Authors:** Muzafar Riyaz

**Affiliations:** 1 Xavier Research Foundation, St. Xavier's College, Palayamkottai, Tamil Nadu, India

The article "The genome sequence of Clancy’s Rustic, Caradrina kadenii" probes into the genetic exploration of Caradrina kadenii, a species belonging to the Lepidoptera order. The research aims to shed light on the genetic makeup of this species, contributing valuable insights into its evolutionary biology and ecological adaptation. The article provides comprehensive visualizations, such as BlobToolKit Snailplot, GC-coverage plot, and Hi-C contact map, offering readers an intuitive understanding of the genome's structural and functional attributes. These visual aids enhance the clarity of the study, allowing readers to grasp the complex genomic data with ease.

 The genome sequence of Clancy’s Rustic, Caradrina kadenii presents a significant contribution to the field of genomics, offering a detailed and well-curated genome assembly of Caradrina kadenii. By elucidating the genetic makeup of this species, the study not only advances our understanding of Lepidoptera evolution but also provides a valuable resource for future research in areas such as ecology, adaptation, and conservation. The rigorous methodology, combined with insightful visualizations, ensures the credibility and relevance of the findings, making this article a noteworthy addition to the genomic research landscape.

Are sufficient details of methods and materials provided to allow replication by others?

Yes

Is the rationale for creating the dataset(s) clearly described?

Yes

Are the datasets clearly presented in a useable and accessible format?

Yes

Are the protocols appropriate and is the work technically sound?

Yes

Reviewer Expertise:

Phylogenomics

I confirm that I have read this submission and believe that I have an appropriate level of expertise to confirm that it is of an acceptable scientific standard.

# Reviewer report: Reviewer response for version 1

**DOI:** 10.21956/wellcomeopenres.21369.r67489

**Published:** 2023-10-10

**Authors:** Lars Höök

**Affiliations:** 1 Department of Ecology and Genetics, Uppsala University, Uppsala, Sweden

The author presents the data note for the genome assembly of the moth C. kadenii.

 The methods are well described and follows established standards for generating high quality chromosome level assemblies.

 The data, as desribed, is of high quality and can be of great value for evolutionary ecology studies due to a recent range expansion of the species. It can further be of value for comparative genomic studies in Lepidoptera.

Are sufficient details of methods and materials provided to allow replication by others?

Yes

Is the rationale for creating the dataset(s) clearly described?

Yes

Are the datasets clearly presented in a useable and accessible format?

Yes

Are the protocols appropriate and is the work technically sound?

Yes

Reviewer Expertise:

Evolutionary genetics

I confirm that I have read this submission and believe that I have an appropriate level of expertise to confirm that it is of an acceptable scientific standard.
